# Antioxidant Status in Patients after Breast Mastopexy and Augmentation

**DOI:** 10.3390/medicina60071046

**Published:** 2024-06-26

**Authors:** Kirils Jurševičs, Eduards Jurševičs, Jeļena Krasiļņikova, Andrejs Šķesters, Anna Lece, Ingus Skadiņš

**Affiliations:** 1 Department of Doctoral Studies, Riga Stradiņš University, LV1007 Riga, Latvia; 2 Clinic of Aesthetic Medicine of Medical Doctor Edward Yurshevich, LV1010 Riga, Latvia; lipex@lipex.lv; 3 Department of Human Physiology and Biochemistry, Rīga Stradiņš University, LV1007 Riga, Latvia; jelena.krasilnikova@rsu.lv; 4 Scientific Laboratory of Biochemistry, Riga Stradiņš University, LV1067 Riga, Latvia; andrejs.skesters@rsu.lv (A.Š.); anna.lece@rsu.lv (A.L.); 5 Department of Biology and Microbiology, Rīga Stradiņš University, LV1007 Riga, Latvia; ingus.skadins@rsu.lv

**Keywords:** oxidative stress, blood antioxidant system, breast augmentation, mastopexy, glutathione peroxidase, selenium, selenium protein P

## Abstract

*Background and Objectives*: Mammary gland surgery has become very common, but there are complications of these operations, including the concept of breast implant illness (BII) in women with silicone gel breast implants (SBI), who suffer from various symptoms such as myalgia, arthralgia, fatigue, fever, dry eyes, or dry mouth. Silicone biomaterials are synthetic polymers that have their own physical and chemical properties and can exert their effect at the site of use and possibly on the general status of the body, causing inflammation and oxidative stress signs. The aim of the study was to examine components of the blood antioxidant system (AOS) of the mastopexy and breast augmentation patients before the operation, on the first post-op day, and 6 months after surgery. *Materials and Methods*: Healthy breast surgery patients (women aged 31 to 60 years without visible pathologies) were selected for the study and formed 2 groups: breast lift—mastopexy without silicone biomaterials (I group, 30 patients) and breast augmentation using silicone biomaterials (II group, 28 patients). All patients underwent standard preoperative tests. Glutathione peroxidase (GPxSe) and gamma-glutamyl transferase (GGT) in blood, selenium (Se), selenium protein P (SelPP), and total antioxidant status (TAS) in plasma were measured as AOS parameters. The concentration of vitamin D was also determined. A total of 174 blood tests were performed. *Results*: Overall, there were no differences in both groups in measured antioxidant system indicators over time; neither changes in objective nor subjective status were observed. However, baseline activity of GPxSe was relatively high but restored to normal values 6 months after surgery. In the mastopexy group, GPxSe decreased from 12,961.7 U/L by 18.9% to 10,513.4 U/L, and in the breast augmentation group, from 15,505.0 U/L by 25.1% to 11,265.5 U/L, which is a decrease of 18.9% and 25.1%, respectively. The patients did not note any complaints; other indicators of standard biochemical tests were within normal limits. *Conclusions*: The two types of surgical interventions, breast mastopexy and augmentation of the mammary glands, do not significantly impact blood AOS and are physiological in nature.

## 1. Introduction

Mammary gland operations are very common in modern plastic surgery. There are works that show complications of these operations, including the concept of breast implant illness (BII) in women with silicone gel breast implants (SBI), who suffer from various symptoms such as myalgia, arthralgia, fatigue, fever, dry eyes, or dry mouth [1,2,3]. Some authors explain this as a genetic feature, autoimmune reactivity of the body, cytotoxicity, macrophage activity, etc. [1,2,3,4].

However, there are no sufficient and systematic studies on the state of the antioxidant system in these patients before and after surgery, as well as in the long-term period. Although there are some papers on parameters of antioxidants and activation of oxidative stress (reactive dismutase I, H_2_O_2_, and reactive oxygen species (ROS)), most of the research was performed in vitro and on models [5,6,7,8]. They do not give a general picture of the complex changes in the antioxidant spectrum. In addition, data have been accumulated on the use of various biomaterials, mainly silicone, in various areas of medicine: operative surgery, ophthalmology [9,10], vascular surgery [11], neurosurgery, orthopedics, as well as plastic and reconstructive surgery.

Silicone biomaterials used in medicine are synthetic polymers that have their own physical and chemical properties, and it is logical to assume that they exert their effect locally, at the site of use, and possibly on the overall homeostasis of the body, like mediators of inflammation and thrombus formation, and cause pathological cellular apoptosis. Some parameters that change during oxidative stress and the associated pathogenesis and pathways are described by Dalle-Donne et al. (2006) [12] and the criteria for change by the American Association for Clinical Chemistry (2006).

It can be assumed that the introduction of a foreign material can initiate the activation of oxidative stress both locally and throughout the body as a whole. Therefore, it is of interest to study possible changes in the body’s antioxidant system during acute stress, i.e., the firstpostoperative day, and over a longer period of time. And perhaps some subjective sensations in patients after mammoplasty and objective symptoms and syndromes currently described as BII are explained by the activation of the oxidative stress system and its complications. So, oxidative stress induced by cytotoxic peroxidation of the lipid membrane changes the structure of cell macromolecules, including arachidonic acid, leukocytes, and others.

Oxidative stress, regardless of its inciting factors—excessive physical and psycho-emotional stress, chemical and physical agents, medications, pain, and various types of diseases—causes changes in the body. Respectively, the physiological balance between pro-oxidants and antioxidants is disrupted, including a sharp shift in the RedOx state in favor of pro-oxidants, as a result of which a state characterized as oxidative stress occurs.

Selenium (Se) is one of the most important trace elements, and it plays an important role in many biological processes, including RedOx, in the human body. Selenium enters the body mostly with food and with additional nutritional supplements, both inorganic compounds (selenate and selenite) and organic compounds (selenocysteine, selenomethionine, and methylselenocysteine). Furthermore, the action of selenium in the body is possible after its involvement in the processes of gene activity. Currently, 25 genes have been discovered that are responsible for the development of selenium proteins, such as selenium-dependent glutathione peroxidase, selenoprotein P, selenoprotein S, selenoprotein W, thioredoxin reductases, 3-iodthyronine deionidases, and a number of other selenoproteins. It can be argued that selenium proteins are involved in practically all life processes in the body: oxidative stress regulation, immune response reactions, inflammatory processes, protection of the endoplasmic reticulum of cells against the effects of reactive oxygen species, acting as antiviral and antibacterial agents, etc. [13,14].

Selenium-dependent glutathione peroxidase is one of the most active and visible selenium proteins. The localization of this antioxidative enzyme in the body, organ systems, and cells is marked with an index of 1–6, for example, GSH-GPx1 in the cytoplasm, GSH-GPx4 (PH) in the mitochondria, nucleus, membranes, and GSH-GPx6 such as GSH-GPx2 secreted. The main role of this antioxidative enzyme in the processes of the body is the neutralization of lipid peroxides and hydroperoxides, but in cooperation with catalase, or in the case of acatalase, the conversion of H_2_O_2_ into H_2_O and O_2_. In addition, in certain situations, GPx can take part in the regulation of proinflammatory cytokines in cells during inflammatory processes [15,16,17].

Different types of silicone materials and their modifications were studied, both the surface textures of the biomaterial itself and after treating the polymer with antibacterial agents’ such as triamcinolone, doxycycline, and others [3,11,18,19,20,21]. But there is no comparison between changes in oxidative stress level and overall homeostasis in several silicone devices and the localization of application. Also, in the available literature, there is no data on the influence of silicone breast biomaterials, used in different age groups, on oxidative stress over a defined period, nor on the comparison of changes in the oxidative activity of the body during various types of esthetic surgery of the mammary gland.

The aim of this research is to examine components of the defense antioxidant system of breast augmentation and mastopexy patients before the operation, then on the first post-op day and 6 months after the operation to find a link between the subjective feelings of the patients and objective findings in the oxidative stress system.

## 2. Materials and Methods

### 2.1. Patients

Two types of breast surgery were used in this study: breast augmentation using silicone biomaterials and breast lift—mastopexy, without the use of silicone biomaterials. 

To achieve patients’ desires for bigger breast volume, breast augmentation was performed with silicone-gel breast implants using an inframammary approach in a subpectoral dual-plane fashion. All the implants were micro-textured. To compensate for breast ptosis, breast mastopexy, or reduction mastopexy, was performed. Mastopexy was conducted in an inverted T fashion, with transposition of nipple–areolar complex (NAC) on the superomedial pedicle. The skin was de-epithelized according to preoperative makings, breast tissue was divided, excessive tissue was removed for better symmetry of the breasts, and the NAC was positioned in a lifted fashion. In this way, the sensation and blood supply for the NAC are not compromised. Specific surgical methods were chosen due to their reliable and predictable esthetic outcomes. 

For this research, healthy women who applied to the Clinic of Aesthetic Medicine of Medical Doctor Edward Yurshevich, Riga, Latvia, for surgical breast correction, aged 31 to 60 years, without visible pathologies, were selected. Their health status and suitability for the operation were thoroughly inspected. A clinical history, blood tests, thorax fluorogram, and ECG were collected. In cases of detected anomalies and general surgical contraindications, the patient was excluded from the surgery as well as the research.

The first group included 30 women aged 31 to 60 years with complaints of unaesthetic and ptotic mammary glands. They were scheduled for a surgical breast lift operation—bilateral mastopexy. None of the patients had any concomitant pathologies at the time of surgery. The second group consisted of 28 women aged 33 to 54 years with complaints of small breast size. Bilateral breast augmentation using silicone implants filled with silicone gel was indicated in the case of these women. At the time of the operation, no concomitant pathologies were identified. 

The operations were carried out in the center of esthetic surgery, and venous blood samples were drawn from each patient in both groups on an empty stomach three times during the study: before the operation, on the first post-operation day (the next day after the operation), and six months after surgery. Blood tests and biochemical studies were carried out in specialized, certified laboratories.

### 2.2. Laboratory Methods

All patients underwent standard preoperative tests, such as a complete blood count, urinalysis with microscopy, infectious disease tests, and the determination of vitamin D concentration. Blood samples were analyzed in the following certified laboratories: Central Laboratory, E. Gulbja Laboratorija, Scientific Laboratory of Biochemistry in the Institute of Occupational Safety and Environmental Health at Riga Stradiņš University—all located in Riga, Latvia.

Several parameters of the antioxidant defense system were determined—selenium-dependent glutathione peroxidase in blood (GPxSe), gamma-glutamyl transferase in blood (GGT), selenium in plasma (Se), selenium protein P in plasma (SelPP), and total antioxidant status in plasma (TAS).

Selenium dependent Glutathione peroxidase is a selenium-dependent enzyme, that catalyzes the breakdown of hydrogen peroxide and lipid hydroperoxides into compounds less harmful to the cell. It was tested in heparinized blood using the Paglia and Valentine method [22]. Reference values are 4171–10,881 U/L.

Gamma-glutamyl transferase (GGT) is an intracellular enzyme that is located mainly in hepatocytes and is involved in the transport of amino acids (glutamyl) across cell membranes. GGT in the blood impacts redox equilibrium both in cells and in blood circulation. Determined by the kinetic reaction method, reference values are ≤36 U/L in females and ≤61 U/L in males.

Selenium (Se) is an essential trace element required for the function of several selenium-dependent enzymes controlling different metabolic activity in organisms: thyroid hormone deiodinases, selenium protein P in plasma, which is associated with vascular endothelial cells, and selenium protein W, which is found in muscles and regulates muscle metabolism. It is a helper for antioxidant enzymes structure and activity, affect the pro-oxidant/ antioxidant balance of the cells [23,24,25,26]. Lowered selenium in plasma correlates with decreasing tolerance to viral infection, induced ROS formation, which leads to oxidative stress. It induces cell and tissue damage [27] and can weaken the immune system in total [28]. Plasma selenium level was determined using the Alfthan fluorometric method, based on the reduction of selenium compounds at an excitation wavelength of 369 nanometers and an emulsion wavelength of 518 nanometers (previously reported [27]). Reference values are 70 to 130 µg/L [29]. 

Selenium Protein P is found in plasma and associated with endothelial cells as well as an antioxidant capable of protecting endothelial cells from reactive hydrogen species—peroxynitrite damage [23,30]. Plasma SelPP levels were measured by a validated commercial SELENOP-specific ELISA kit according to the instructions of the supplier. Reference values are 2.56 to 6.63 mg/L.

Total antioxidant status was determined in plasma. This is a combination of all the antioxidant components of scavengers and “transformers” of free radicals—oxide, superoxide, hydroperoxide, nitrite, lipoperoxide, and aggressive biomolecules that non-specifically destroy cell membranes and intracellular structures. That can cause progressive processes of pathological apoptosis and chronic inflammation of tissues and organs. The photometric method was used, reference units are 1.3–1.77 mmol/L.

Chemicals were produced by Sigma-Aldrich (Spruce St., Saint Louis, MO, USA).

Data were calculated and analyzed using Microsoft Excel Professional Plus 2405 and IBM SPSS 22, using descriptive statistics as well as a non-parametric Mann–Whitney U test with a significance level of *p* < 0.05.

## 3. Results

**Figure 1:**
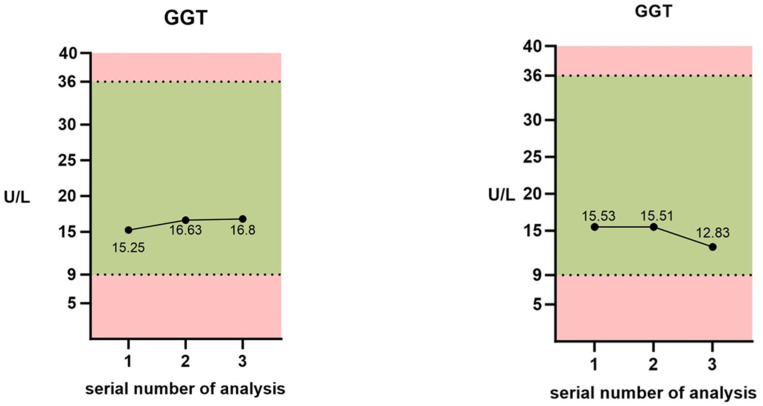

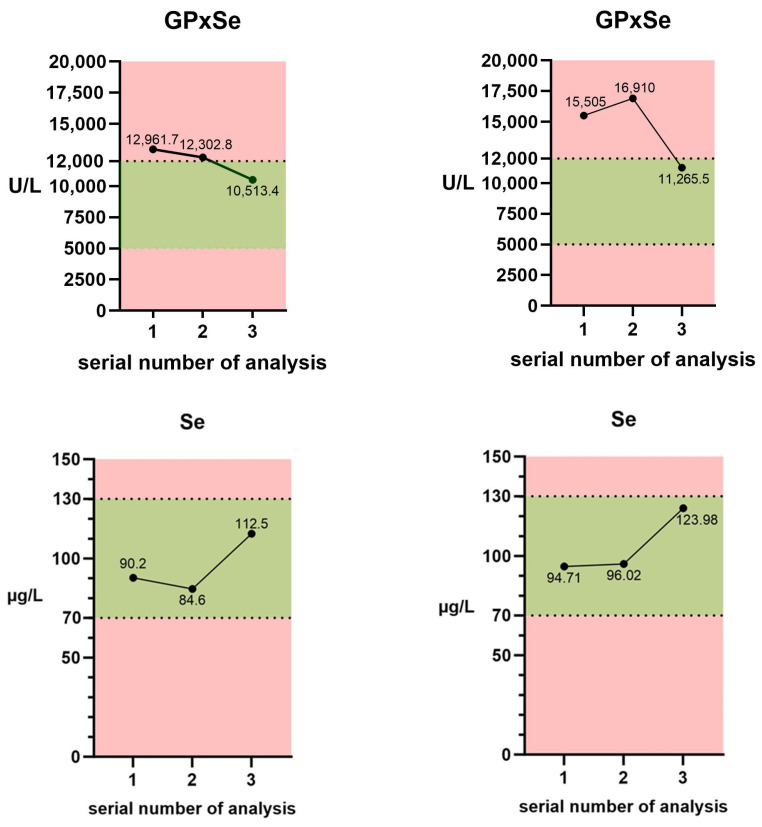

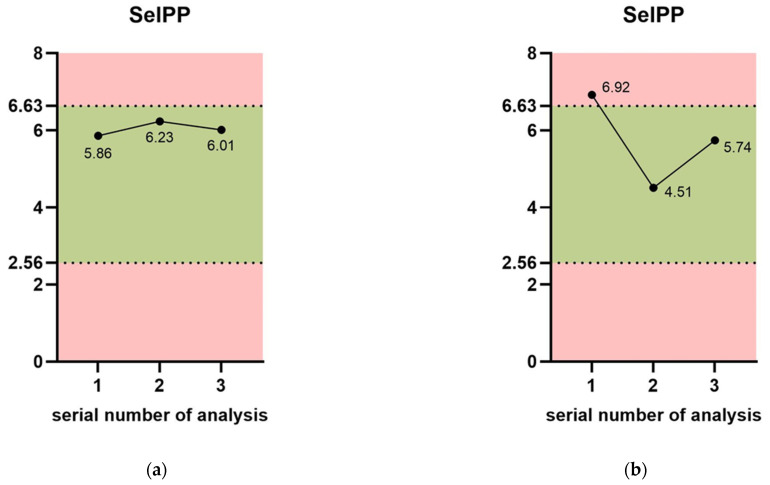
Changes in the measured parameters of the blood antioxidant defense system: 1—before the operation, 2—on the next day after the operation, 3—six months after surgery. Normal values are presented in green. (**a**) Mastopexy; (**b**) breast augmentation. GGT—gamma-glutamyl transferase; GPxSe—selenium dependent glutathione peroxidase; Se—selenium; SelPP—selenium protein P.

After 174 tests, the following results were obtained: In the group with mastopexy, when assessing the effect of oxidative stress on the activity of the antioxidant system, dynamic changes were observed in all indicators of the enzyme link (GPxSe, GGT) in the “helpers” group (Se in plasma), and according to the ESP EN 1980 classification in the “various” group (SelPP), with normal values highlighted in light green (Figure 1a,b).

The average GPxSe value on the first day after surgery decreased by 5.9%, which means that it approached the physiological norm. After 6 months, it decreased by 18.9% compared to the baseline and corresponded to normal values. When data were analyzed individually, in the early stages, the level of GPxSe decreased in those patients in whom this indicator was significantly higher than normal. This difference was 16.4%. Long-term results in this group were lower by 30.1% compared to the baseline and corresponded to 10,368.75 U/L, which is within normal values. Thus, we can talk about the restoration of the homeostatic state of the cell in response to the oxidative stress caused by mastopexy. The relatively high level of antioxidant enzymes can be explained by the psycho-emotional stress of the patient before the operation, since the indicator of biochemical clinical status and analytical follow-up did not detect any pathology. This obviously explains the normalization of this parameter six months later—a full period of restoration of the usual lifestyle. In acute periods, changes in GPxSe may be associated with surgical intervention; this is consistent with the data of P. Vijayalakshmi [31]. 

On the other hand, J. Romero et al. [32] described that silicon polymer materials have an effect on lipid peroxidation (LPO) in the liver and induce ROS formation. LPO products show vascular effects in different zones. Long-persisting aldehydes and hydroperoxides spread from their site of origin and by blood circulation attack targets distant from the initial radical event.

During breast augmentation, the following changes in antioxidant enzyme defense were observed (Figure 1b). It was statistically significant that GPxSe before surgery was higher than normal in 75% of patients and was 16,857 U/L on average. Prior to the surgery, this indicator increased to 15,505.1 U/L in the entire group of subjects, which was also statistically significant. Obviously, this change in GPxSe before surgery may be associated with the activation of intracellular protective mechanisms using the GPxSe pool inside the cell and disruption of the permeability of cell membranes, which leads to the release of the enzyme into the bloodstream. This corresponds to its increase when determined in blood plasma, and perhaps this is explained by the presence of a preoperative subjectively stressed state, the occurrence of which induces oxidative stress, as described above.

The long-term presence of silicone biomaterials locally can cause morphological changes in a degenerative nature, tissue calcification, oxidative stress during subcutaneous implantation in vitro and in vivo, as well as upregulate hypoxia induced factor-1 alfa (MIF-1alfa), an increase in free H_2_O_2_ peroxides, super-expressed superoxide dismutase (SOD-1), catalase (Cat-1), nitric oxide synthase-3 (NOS_3_), and autophagy [8,33,34].

As a reactive response to surgery on the first post-operation day, GPxSe increased by 8.7% and 9.1%, respectively, in cases with elevated and normal GPxSe levels before breast augmentation. Six months later, the values in these groups decreased by 32.6% and 27.3%, respectively, i.e., more with the initial disruption of antioxidant homeostasis, and the GPxSe level returned to normal limits of 11,265.5 U/L to 11,361.3 U/L. In the long-term period, the result of physiological action and normalization of cellular metabolism after breast augmentation is obvious. This is consistent with the improvement in the objective status of patients. In comparison to GPxSe in patients with mastopexy, where the level of GPxSe in blood decreased both on the first post-op day and six months later, in the augmentation group, there were more pronounced dynamics and restoration of the normal level of cellular antioxidant activity.

The next enzyme indicator—GGT—was in the normal range before surgery, as well as on the first day and in the long-term period. After surgery, a slight increase in transferase was observed in both groups, 8.5% and 9.8%, respectively. This correlates well with the dynamics of GPxSe activity: possible activation of the resynthesis of intracellular glutathione. Activation and an increase in glutathione levels generally increase antioxidant capacity and resistance to oxidative stress in many types of pathology and acute conditions [35]. In the long-term period of the breast augmentation group, GGT decreased by 17.4%, but was within normal limits throughout the study. 

When assessing non-enzymatic antioxidant defense systems, the following was revealed: in all patients with mastopexy in all stages of laboratory examination, the level of selenium in plasma was within normal limits, but there were changes in dynamics. On the first post-op day, selenium decreased by 15.4% and six months later increased by 24.7%. The selenium transport protein SelPP also corresponded to normal values and increased by 6.3%, which matches changes in the level of GPxSe.

In the breast augmentation group, the levels of Se and SelPP in all periods of the study were within normal values. In the long term, Se increased by 30.9%, and SelPP decreased by 35%. This may reflect the dynamics of GPxSe activation and glutathione metabolism. The obtained data on the participation of Se and SelPP in the processes of restoring homeostasis and immunoprotective and antioxidant activity during oxidative stress are consistent with the data of A Šķesters, A. Lece et al. 2023 [36], E. Mangiapane et al. 2014 [37], and A. Moghaddam et al. 2020 [38].

It should be noted that the level of total vitamin D in the blood of all patients throughout the study was below normal by an average of 23.8%, while the initial level of vitamin D in both groups ranged from 32.5 to 34.3 mg/L. After surgery, in patients with mastopexy, vitamin D levels slightly increased by 3.69%, and in the group with augmentation, by 6.99%.

During the entire observation period, the objective and subjective status of the patients were normal. There were no side effects or complications observed; the patients’ well-being and mood were consistently positive, both during their hospital stay and during outpatient observation.

Regardless of the initial data on the antioxidant status, there were no differences in indicators over time in the group with mastopexy; neither change in objective nor subjective status was observed, and similarly in the group with augmentation. The patients did not note any complaints, and other indicators of standard biochemical tests were within normal limits.

## 4. Discussion

Some papers on a complete study of vitamin D in its active form, 12.25 dihydroxy-vitamin D3, have appeared in recent years. On its therapeutic and preventive effect against oxidative stress, as well as superoxide anions, reducing the phenomenon of pathological apoptosis by blocking the extrinsic cascade by positively controlling phosphor active ERKs level, MEKs/ERKs inhibitor UO 126 reversed the vitamin D antioxidant effect [39,40]. The hormonal form of vitamin D, calcitriol, has antiproliferative properties, decreases tumor necrosis factor-alfa (TNF-alfa), and cytokine-induced and programmed cell death by caspase-dependent and caspase-independent pathways. As well as preventing mitochondrial injury associated with a decrease in mitochondrial potential, which results in the secondary excessive ROS generation of proteins involved in caspase activation and programmed cell death [41,42].

Some papers show the antioxidant role of D3 (1.25 (OH)2 D3) against oxidative stress caused by muscle proteolysis, with glutathione-dependent enzyme activation and decreasing of SOD and catalase enzymes in muscle cells. Moreover, vitamin D3 enhances SOD, catalase, and GPxSe in various injuries of hepatic, pancreatic, and renal cells [43,44].

This is consistent with our data showing an increase in initial GPxSe in plasma due to an obvious decrease in the enzyme inside cells with vitamin D3 deficiency in the background. All patients in the group with augmentation and mastopexy initially had an indicator of the total state of antioxidant parameters of the enzyme and non-enzyme components (vitamins, trace minerals, microelements, some protein fractions, etc.). TAS is slightly lower than the norm, 1.74, with the normal values being 1.77 to 2.22. In both groups on the first day after surgery and in the long-term period, it fluctuated from 0.58% to 3.8%, which was not significant. And, on the other hand, it shows the good compensatory capacity of the antioxidant system for oxidative stress that occurs during these types of plastic surgeries.

It should be mentioned that our study has a prospective design, which of course has its default limitations, but the main issue of interrupted follow-up did not occur because the patients were responsible and interested in the results.

Understanding the processes caused by breast surgery on a cellular level can improve recommendations during the healing and rehabilitation process. This, on the other hand, can speed up the recovery and improve the overall quality of life. Continuous research is needed in this field. Various breast surgery operations, both esthetic and reconstructive, can be studied in the future.

## 5. Conclusions

In the presented work, the following results and patterns were revealed: In the group of patients with mastopexy in the acute period, enzymes with antioxidant activity (GPxSe) decreased on average by 5.9% and six months after surgery by 18.9%. At the same time, in patients with initially elevated enzyme levels, a decrease in GPxSe was observed on the first day after surgery by 16.4% and a restoration to normal, a decrease of 30.1%. In the group of patients with breast augmentation, all women had an increased level of GPxSe (16,522 to 17,430 U/L). After surgery, the enzyme level in plasma increased on the first day by 9.1%, but after six months it decreased by 27.34–32.6% and was within normal values. There were no significant changes in the dynamics of GGT levels, but with breast augmentation, the amount of plasma transferase decreased by 17.4%.

The non-enzymatic link of antioxidant defense systems, Se and SelPP, in blood plasma were within normal limits throughout the entire study period, and after 6 months, they increased by 20.7% in the group with breast augmentation and 6.3%, respectively, in the group with mastopexy. In patients with augmentation, the Se level increased by 30.9% but did not exceed normal values. Its transport form in plasma, SelPP, decreased by 35% and returned to normal. The total indicator of the activity of antioxidant defense systems (TAS) did not change throughout the study. Thus, based on the demonstrated dynamics of long-term monitoring of the results of clinical and biochemical studies, it can be claimed that both types of surgical interventions described are physiological in nature. Thus, based on the shown dynamics of long-term monitoring of the results of clinical and biochemical studies, it can be claimed, that both types of surgical interventions described are physiological in nature. The mentioned operations on the breast are not only physiological in nature from a cellular point of view but also provide significant relief and social and economic benefits, as well as improve the overall quality of life for patients who desire these operations.

## Data Availability

Data are contained within the article.
